# Evaluation of Whether Emergency Physicians Should Join the Multidisciplinary Team for Older Hip Fracture Patients

**DOI:** 10.3389/fsurg.2022.842978

**Published:** 2022-05-18

**Authors:** Lan Guan, Cong Wang, Bin Zhao, Minghui Yang, Shiwen Zhu, Xinbao Wu

**Affiliations:** ^1^ Department of Emergency, Beijing Jishuitan Hospital, Beijing, China; ^2^ Department of Orthopedics and Traumatology, Beijing Jishuitan Hospital, Beijing, China

**Keywords:** older people, hip fracture, emergency physician, multidisciplinary co-management, China

## Abstract

**Background:**

Geriatric hip fracture is one of the most common end-stage events in older patients with osteoporosis. We aimed to improve the original co-management process by engaging emergency physicians in the preoperative multidisciplinary management team (MDT). We evaluated this intervention in terms of reducing patient waiting time before surgery.

**Methods:**

Emergency Department data and hospitalization data for patients diagnosed with geriatric hip fractures in Beijing Jishuitan Hospital (JSTH) were collected and sorted into the intervention group, for whom the MDT included emergency physicians (from January 2019 to December 2019), and the control group (from January 2017 to December 2017). The percentage of patients treated with surgery within 48 h of admission was used as the primary outcome. The secondary outcomes included the time from emergency visit to admission (hours), the time from admission to discharge (days), the percentage of patients receiving surgical treatment after admission, the rate of perioperative medical complications during hospitalization, postoperative admission to the Intensive Care Unit, and total deaths during hospitalization.

**Results:**

A total of 2,152 patients were enrolled. The rate of hypertension (58.5% vs 52.1%), coronary heart disease (24.6% vs 19.9%), and cerebrovascular disease (19.4% vs 15.5%) was higher in the intervention group than in the control group. The percentage of patients receiving surgical treatment in the intervention group (98.3%) was significantly higher than in the control group (96.3%, *p* = 0.004). The proportion of patients receiving surgical treatment within 48 h of admission was significantly higher in the intervention group (82.4%) than in the control group (60.4%, *p* < 0.001). The hospital stay was significantly shorter in the intervention group compared with the control group (*p* < 0.001). The incidence of perioperative medical complications and mortality during hospitalization was similar in the two groups.

**Conclusions:**

Involving emergency physicians in the MDT can reduce the waiting time before surgery and the hospital stay for older hip fracture patients.

## Introduction

Geriatric hip fracture is one of the most common end-stage events in older patients with osteoporosis. Hip fracture in older people can result in the loss of mobility and independence and even death (1, 2). The most common hip fractures are femoral neck fractures, intertrochanteric fractures, and subtrochanteric fractures. According to the International Osteoporosis Organization, the number of hip fractures in the world will reach 6.26 million by 2050, with more than 50% occurring in Asian populations (3). By 2050, it is expected that there will be 1.4 billion Chinese, with 365 million aged 65+, a number representing 26.1% of the country’s total population (4). With the increasing economic and social burden of geriatric hip fractures in developing countries, optimization of existing clinical processes to be able to offer cost-effective and high-quality medical services to older patients with hip fractures is urgently warranted.

In recent years, we have seen a development in orthogeriatric care, which can be defined as the collaboration between orthopedic surgeons and geriatricians to improve hip fracture patient outcomes during hospital admission. On the basis of this concept, it has been generally recognized that the multidisciplinary management team (MDT) model can shorten the preoperative waiting time, reduce the length of hospital stays, and improve the efficiency of surgical treatment for older hip fracture patients (5, 6). However, models of orthogeriatric co-management currently in use worldwide are different in the composition of joint expert group and the time, space and frequency of ward rounds (7, 8). These include the participation and cooperation of experts from different related disciplines and departments, such as anesthesiologists, rehabilitation doctors, emergency doctors, and general practitioners (8, 9). The benefits of anesthesiologists participating in a MDT with orthopedic surgeons and geriatricians have been most discussed in the literature (9–11).

In order to best leverage the academic advantages of our hospital and optimize management of older hip fracture patients in our country, we implemented a co-management program in 2015. Our program was based on the best practice guidelines for hip fractures in the United Kingdom (UK) (12). Hospitalized patients were jointly managed by orthopedic doctors and geriatricians (13), with treatment plans led by an orthopedic surgeon, and auxiliary diagnosis and treatment performed by a geriatrician. We previously reported that implementation of this co-management model resulted in significantly increased in the number of patients received surgery within 48 h of hospitalization (6.4% before implementation of the co-management model versus 50% with the model). This co-management model maintained a stable process and team from January 2017 to December 2017 at Beijing Jishuitan Hospital (JSTH). While using a MDT has greatly improved our practice in the past few years, certain gaps in patient outcomes were seen compared to the findings from the UK. The benchmark set in the UK guidelines was 83%. We further analyzed the reasons for this gap and found that there was an imbalance relationship between limited number of beds in the orthogeriatric co-management ward and high volume of patients visits for treatment in JSTH. This leads to prolong patients’ waiting time in the emergency department (ED). However, due to the policy and the space capacity constraints, it was not possible to increase the number of beds in a short term. In response to this situation, our research team further amended the joint management model by engaging emergency physicians to the original MDT for older hip fracture patients in 2018. This new joint management model was an improved multidisciplinary collaboration model with a stable process and team from January 2019 to December 2019 at JSTH.

To our knowledge, this retrospective study is the first to discuss, as an intervention factor, the contributions of emergency physicians in the MDT for older patients with hip fractures. We hope that these data will provide guidance and help to further optimize management process for these patients.

## Materials and Methods

### Study Design

This was a single-center retrospective study. Data were collected from the emergency and inpatient electronic medical record of all older patients (age ≥ 65 years) diagnosed with hip fractures and admitted to Beijing JSTH for treatment from January 2019 to December 2019 (intervention group) and January 2017 to December 2017 (control group) respectively. In addition, we increased the number of beds in our orthogeriatric co-management ward from 13 to 18 in 2018. This study was approved by the Ethics Committee of the Beijing JSTH (No. 202109-61).

### Study Site and Participants

Beijing JSTH is a leading national orthopedic hospital in China and the fourth medical college of Peking University. It has approximately 1,500 beds and performs over 40,000 orthopedic surgeries every year. Patients were included if they fulfilled the following criteria: (1) Age ≥ 65 years old; (2) Hip fractures (including femoral neck fractures, intertrochanteric fractures and subtrochanteric fractures) diagnosed according to the International Classification of Diseases Code (ICD-10); (3) Diagnosis and treatment within 3 weeks of the time of injury. We excluded patients if: (1) age < 65 years old; (2) injury time exceeded 3 weeks; (3) pathological fractures; (4) combined with fractures at other locations (such as pelvic fractures, upper limb fractures, and clavicular fractures).

### Intervention

The intervention was the addition of emergency physicians into the original MDT for older patients with hip fracture. The following paragraph describes the specific implementation processes of the two co-management models before the intervention and after the intervention.

**Figure 1:**
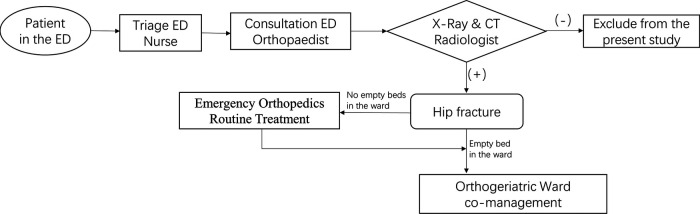
Flow chart of operation mode of control group.

In the control group (Figure 1), each patient was diagnosed with hip fracture after the emergency orthopedic surgeon routinely completed the relevant examination in the ED. All patients eligible for surgery were admitted to the orthogeriatric co-management ward. The management of all admitted patients followed the “orthopedic surgeon + geriatrician” joint management model, and the whole process was led by the orthopedic doctor. In this plan, the ward's anesthesiology and rehabilitation teams participated in the patient's hospital stay. This collaboration covered preoperative management (including pain management, preoperative assessment, and optimization) and postoperative management (including early mobilization, complications prevention, falls assessment, osteoporosis management, and physiotherapy).

**Figure 2:**
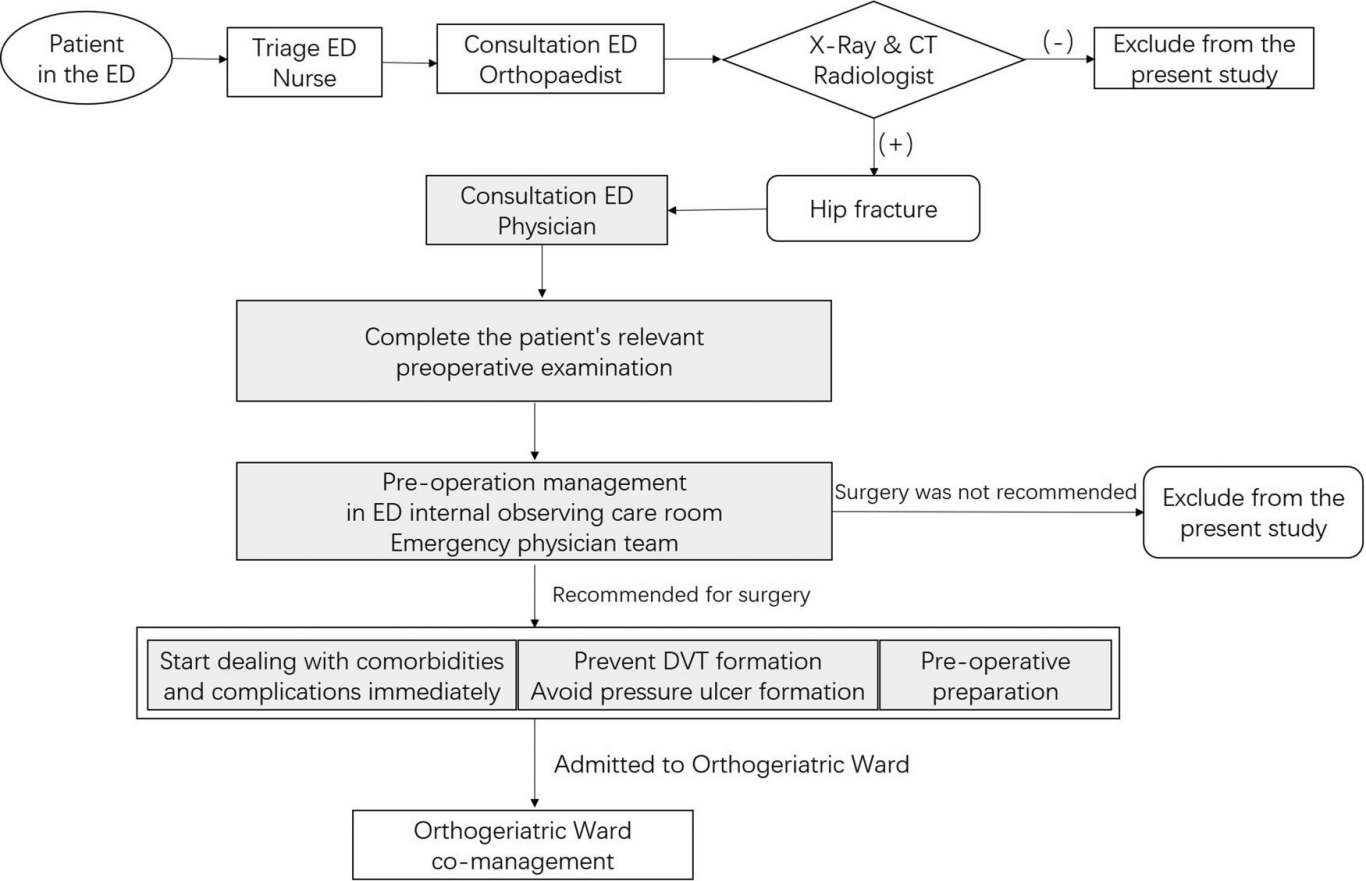
Flow chart of operation mode of intervention group.

In the intervention group mode (Figure 2), emergency physicians in the ED routinely participated in the reception, clinical examination and diagnostic work-up of all older patients diagnosed with hip fracture by the emergency orthopedic surgeon. The emergency physicians immediately started the diagnosis and treatment of each patient’s underlying disease and comorbidities based on the patient history and clinical examination. Management during this period included analgesia, anti-microbials, correction of water and electrolyte disturbances, control of blood pressure, control of blood sugar, improvement of oxygenation, maintenance of unobstructed urine and feces, anticoagulation, prevention of deep venous thrombosis of lower limbs, improvement of coronary blood supply and cerebral blood flow, correction of severe anemia, volume management, correction of arrhythmia, and nutritional support. The remaining procedures were the same as in the control group.

The shaded sections in the flowchart were interventions.

### Data Collection

The emergency data and hospitalization data were collected from the electronic medical record database of JSTH, including the fracture types of the two groups of older patients with hip fractures, underlying diseases (including hypertension, coronary heart disease, diabetes, cerebrovascular disease, lung diseases, and malignant tumors), injury time, emergency visit time, admission time, discharge time, whether surgical treatment was performed, surgery start time, surgical procedure, perioperative medical complications during hospitalization (including chest infection, urinary tract infection, cerebral infarction, acute coronary syndrome, deep vein thrombosis, and pulmonary embolism), postoperative admission to the ICU, and death during hospitalization.

### Outcomes

The primary outcome was the percentage of patients treated with surgery within 48 h of admission.

Secondary outcomes included the time from emergency visit to admission (hours), the time from admission to discharge (days), the percentage of patients receiving surgical treatment after admission, the rate of perioperative medical complications during hospitalization, the rate of postoperative ICU admissions, and the rate of total deaths during hospitalization.

### Statistical Analysis

The data was analyzed statistically by IBM SPSS Statistics 21.0 and *p* < 0.05 was considered statistically significant. Normally distributed data were expressed as the mean ± standard deviation (SD), and the independent sample *t*-test was used for comparisons between the two groups; data that was not normally distributed were expressed as the median (interquartile range) and the Mann-Whitney U test was used to compare the two groups. Count data was described by rate or composition ratio, and the two groups were compared by the chi-square test or the Fisher’s exact test.

## Results

A total of 2,152 patients were enrolled in this study, with 1,282 patients in the intervention group (mean age 79.7 ± 7.9 years; 28.1% males) and 870 patients in the control group (mean age 79.7 ± 7.3 years; 30.2% males). Of those 1,282 patients in the intervention group, there were 666 femoral neck fractures (52.0%), 587 femoral intertrochanteric fractures (45.8%) and 29 (2.3%) femoral subtrochanteric fractures. Fractures in the control group patients included 435 femoral neck fractures (50.0%), 419 femoral intertrochanteric fractures (48.2%), and 16 (1.8%) femoral subtrochanteric fractures.

There were no statistical differences between the two groups of patients in terms of sex, age, whether the emergency visit to Beijing JSTH occurred on the same day as the injury, fracture type and surgical procedure.

We compared the underlying diseases in the two groups of patients. The percentage of patients with hypertension (*χ*^2^ = 8.702, *p* = 0.003), coronary heart disease (*χ*^2^ = 6.490, *p* = 0.011), and cerebrovascular disease (*χ*^2^ = 5.393, *p* = 0.020) was higher in the intervention group (58.5%, 24.6%, 19.4%, respectively) than in the control group (52.1%, 19.9%, 15.5%, respectively). There was no significant difference in the incidence of diabetes, lung diseases (chronic obstructive pulmonary disease, bronchial asthma, bronchiectasis, tuberculosis, or silicosis) or malignant tumors between the two groups (Table 1).

**Table 1:** Comparison of baseline information between the two groups of patients.

Varibles	Total number of cases	Intervention group	Control group	*p*-value
Sex				
Male	623	360 (28.1%)	263 (30.2%)	0.281
Female	1,529	922 (71.9%)	607 (69.8%)	
Age (years)	2,152	79.7 ± 7.9	79.7 ± 7.3	0.840
Emergency visit of JSTH on the same day after injury				
No	951	585 (45.6%)	366 (42.1%)	0.102
Yes	1,201	697 (54.4%)	504 (57.9%)	
Fracture type				
Femoral neck fracture	1,101	666 (52.0%)	435 (50.0%)	0.484
Intertrochanteric fracture of femur	1,006	587 (45.8%)	419 (48.2%)	
Subtrochanteric fracture of femur	45	29 (2.3%)	16 (1.8%)	
Surgical procedure				
Hemiarthroplasty	670	411 (32.6%)	259 (30.9%)	0.569
Cannulated screw fixation	229	132 (10.5%)	97 (11.6%)	
Total hip arthroplasty	187	118 (9.4%)	69 (8.2%)	
Nail fixation	1,001	594 (47.1%)	407 (48.6%)	
Plate and screw fixation	11	5 (0.4%)	6 (0.7%)	
Underlying disease				
Hypertension				
No	949	532 (41.5%)	417 (47.9%)	0.003
Yes	1,203	750 (58.5%)	453 (52.1%)	
Coronary heart disease				
No	1,664	967 (75.4%)	697 (80.1%)	0.011
Yes	488	315 (24.6%)	173 (19.9%)	
Diabetes mellitus				
No	1,601	936 (73.0%)	665 (76.4%)	0.074
Yes	551	346 (27.0%)	205 (23.6%)	
Lung disease				
No	1,964	1,160 (90.6%)	804 (92.4%)	0.148
Yes	186	120 (9.4%)	66 (7.6%)	
Cerebrovascular disease				
No	1,768	1,033 (80.6%)	735 (84.5%)	0.020
Yes	384	249 (19.4%)	135 (15.5%)	
Malignant tumor				
No	2,047	1,221 (95.2%)	826 (94.9%)	0.752
Yes	105	61 (4.8%)	44 (5.1%)	

### Primary outcome

**Figure 3:**
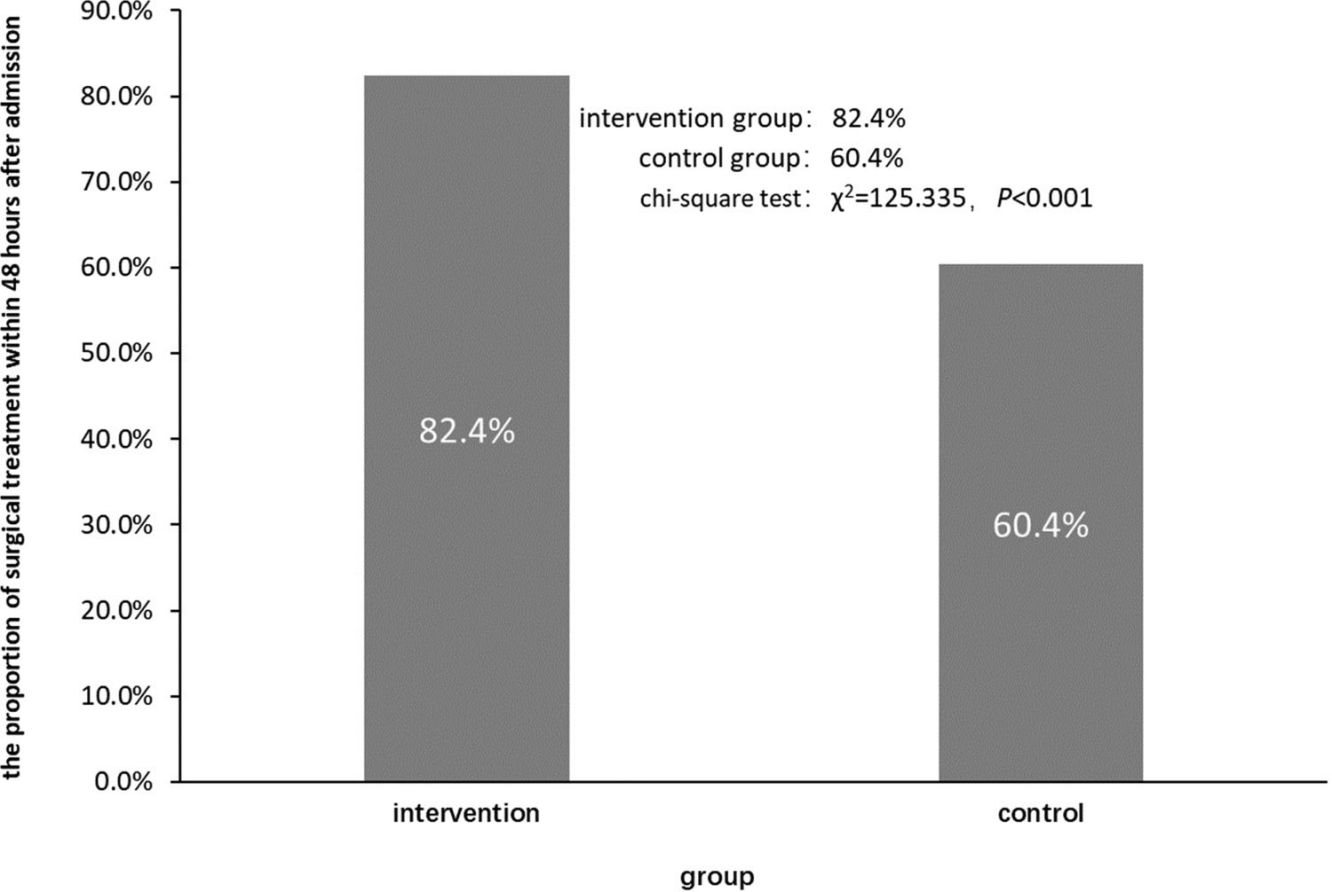
Comparison of the proportion of surgical treatment within 48 h after admission between the two groups.

In this study, the percentage of patients in the intervention group receiving surgical treatment within 48 h of admission was 82.4% (1,038/1,260), which was significantly higher than in the control group (60.4%, 506/838, *χ*^2^ = 125.335, *p* < 0.001, Figure 3).

### Secondary Outcomes

The time from emergency visit to admission and the time from admission to discharge in the two groups of patients is shown in Table 2.

**Table 2:** Comparison of time between the two groups.

	Intervention group (*n* = 1,282)	control group (*n* = 870)	*p*-value
Hours from emergency to admission	20.0 (15.0, 25.0)	20.2 (6.3, 43.7)	0.748
Days from admission to discharge (median, IQR)	4.0 (3.0, 5.0)	5.0 (4.0, 5.0)	<0.001
Days from admission to discharge (category)			
<3.0	549 (42.8%)	99 (11.4%)	<0.001
3.0–4.9	555 (43.3%)	573 (65.9%)	
5.0–6.9	142 (11.1%)	150 (17.3%)	
≥7.0	36 (2.8%)	47 (5.4%)	

There was no significant difference (*Z* = −0.322, *p* = 0.748) in the time from emergency visit to admission between patients in the intervention group (20.0 h (15.0, 25.0)) and patients in the control group (20.2 h (6.3, 43.7)).

The time from admission to discharge was significantly shorter in the invention group (4.0 days (3.0, 5.0)) than in the control group (5.0 days (4.0, 5.0), *Z* = −14.715, *p* < 0.001). When the days from admission to discharge were stratified into categories according to days, there were significantly more patients stayed for less than 3 days in the intervention group (42.8%) than in the control group (11.4%).

The percentage of patients who received surgery after admission in the two groups is shown in Table 3. More patients in the intervention group (98.3% (1,260/1,282)) received surgery, compared with the control group (96.3% (838/870), *χ*^2^ = 8.156, *p* = 0.004).

**Table 3:** Comparison of surgical treatment mode between the two groups.

	Total number of cases	Intervention group	Control group	*p*-value
Operation	2,098	1,260 (98.3%)	838 (96.3%)	0.004
Conservative	54	22 (1.7%)	32 (3.7%)	

The rates of perioperative medical complications during hospitalization, postoperative admission to the ICU, and total mortality during hospitalization in the two groups is shown in Table 4. The incidence of perioperative medical complications during hospitalization were not significantly different in the intervention and control groups (23.8% (305/1,282) and 24.9% (217/870), respectively, *χ*^2^ = 0.374, *p* = 0.541).

**Table 4:** Comparison of the perioperative medical complications during hospitalization, postoperative ICU admission and total mortality during hospitalization between the two groups.

	Total number of cases	Intervention group	Control group	*p*-value
Perioperative complications				
No	1,630	977 (76.2%)	653 (75.1%)	0.541
Yes	522	305 (23.8%)	217 (24.9%)	
Postoperative ICU admission				
No	1,829	1,067 (84.7%)	762 (90.9%)	<0.001
Yes	269	193 (15.3%)	76 (9.1%)	
Death in hospital				
No	2,145	1,280 (99.8%)	865 (99.4%)	0.094
Yes	7	2 (0.2%)	5 (0.6%)	

However, a significantly higher percentage of patients in the intervention group were admitted to the ICU after surgery, compared with the control group (15.3% (193/1,260) and 9.1% (76/838), respectively, *χ*^2^ = 17.578, *p* < 0.001).

The total death rates during hospitalization were not significantly different between the intervention group and control group (0.2% (2/1,282) and 0.6% (5/870), respectively, *χ*^2^ = 2.802, *p* = 0.094).

## Discussion

The present study evaluated the effect of the addition of emergency physicians into the original MDT for older patients with hip fracture in the China’s leading orthopedic hospital, which significantly increased the proportion of patients receiving surgery within the timeframe of 48 h, as recommended in the Blue Book guidelines. There was also a significant decrease in the time from admission to discharge, and the time from injury to discharge was also observed.

Orthogeriatric co-management models of care have medical and economic advantages (14–16). Numerous studies have confirmed that the multidisciplinary team model can reduce preoperative waiting time, shorten hospital stay and improve clinical outcomes (5–8, 17). However, it is not clear which of the various multidisciplinary co-management models has the best patient outcomes. Each study has slight different multidisciplinary co-management model. A co-management system should be tailored to each site and country based on preexisting infrastructure and resources. Most of these studies suggest that co-management model can effectively reduce the rates of perioperative complications, short-term mortality and long-term mortality in older patients with hip fractures (17–20); whereas some studies suggest no, or uncertain, improvement in these outcomes (21–23). Despite several models have been published, there are still no clear recommendations on organization of multidisciplinary co-management team, and no guidelines on further changes in the standard of care that might be needed for this purpose. Preliminary work by our research group showed that joint management by orthopedic doctors and geriatricians had positive results for older patients with hip fractures that was half of the patients who received co-management received surgery within 48 h of ward admission compared to 6.4% previously (13). Multidisciplinary co-management has great potential for scaling across the entire Chinese population (24).

In the UK, there is an requirement that patients should to be admitted to the inpatient ward within four hours of their emergency visit (reference the UK Blue Book), and therefore, patients stay in the emergency department for a relatively short amount of time. While in China, there is an imbalance between the number of patients needs to be treated and the number of beds in orthogeriatric co-management wards, which leads to longer stay in the ED and delayed treatment. We urgently wanted to find a solution to this problem. Emergency physicians have unique expertise in the identification and treatment of emergent and critical illnesses. The American College of Emergency Physicians (ACEP) believes that emergency physicians play a central role in the care of injured patients within the healthcare system, either individually or as members of multidisciplinary trauma teams (25). Emergency physicians play an instrumental role in the management of severely injured trauma patients, particularly in the aspects of assessment, resuscitation, airway management, point-of-care ultrasonography, and bedside procedures. ACEP acknowledges the role of trauma surgeons as the providers of definitive care for the most critically injured patients and the importance of close collaboration between emergency physicians and trauma physicians in developing safe systems of care. In fact, the experience of emergency physicians in dealing with emergent conditions in older patients is beyond doubt, and their professional experience may provide new ideas and solutions to improve the organizational structure of existing multidisciplinary teams. Several previous studies have only considered the emergency department as a whole when participating in the process of managing patients (6, 7, 14). To our knowledge, our study is the first to evaluate, as an independent intervention factor, the participation of emergency physicians in a multidisciplinary team managing older patients with hip fractures in China.

Hip fractures in older patients are associated with high morbidity, mortality, and disability rates (26). Surgical treatment is the first choice for older hip fracture patients, and has obvious advantages over conservative treatment (27). Reduced mobility predisposes older patients with hip fractures who wait for an extended period of time for surgical treatment to a variety of complications, including acute cerebral infarction, pressure injury, deep vein thrombosis of the lower limbs (28), and lung and urinary tract infections caused by prolonged bed rest.

Undergoing surgery within 48 h of admission can significantly improve the clinical prognosis of older hip fracture patients (29). The most significant risk factor in older patients with hip fractures is known as the presence of multiple comorbidities, which are more common in older people than in younger patients. More than 40% of older hip fracture patients have hypertension, 14% have chronic lung disease, and 12% have diabetes. Chronic conditions such as diabetes, peptic ulcers, fluid and electrolyte disturbances, psychosis, coagulopathies, renal failure, and paralysis are associated with significant socioeconomic costs (30). Approximately 70% of older patients with hip fractures are graded III to IV in the American Association of Anesthesiologists (ASA) grading system (31). Babette’s study showed that shared decision-making with geriatricians during the acute assessment of, and communication with, older hip fracture patients in the ED leads to significantly more patients and their representatives choosing non-surgical management and palliative care (32). Severe dementia and poor prior function were the most common reasons not to have surgery.

In our study, we found that a significantly higher percentage of patients in the intervention group, compared with the control group, had underlying disease comorbidities, including hypertension, coronary heart disease, and cerebrovascular disease. This was also the most likely reason for the increased percentage of patients in the intervention group admitted to the ICU after surgery.

Emergency physicians and geriatricians have different expertise, and they also have different perspectives in the recognition and understanding of emergent and critical illness. Emergency physicians have earlier direct contact with patients and their families. We routinely brought emergency physicians into our MDT to participate in the preoperative assessment and preparation of older patients with hip fractures. By including emergency physicians in decision making, a higher proportion of patients with comorbidities were considered eligible for surgery and the proportion of hip fracture in-patients treated with surgery was significantly increased. This shows that co-management by emergency physicians and geriatricians has a beneficial effect on the final implementation of surgical treatment for older patients with hip fractures. It also shows that doctors from two specialties, working together with other team members, can complement each other in providing medical care to patients in different locations and at different times.

This process improvement helped us to achieve the desired primary outcome, which was to increase the proportion of older hip fracture patients receiving surgery within 48 h of admission. The length of stay in the ED was not prolonged by including emergency physicians in the MDT. In fact, the overall length of hospital stay was reduced, which is consistent to previous study that a multidisciplinary hip fracture care model could shorten patients’ length of stay and also cost-effective (33).

Emergency physicians, as the first team members providing in-hospital treatment to older hip fracture patients, are best equipped to start a complete preoperative evaluation and preoperative preparations for older patients with hip fractures. Our data show that this intervention is feasible and has positive results. In the initial stage of project implementation and improvement, we received significant assistance from relevant hospital management departments and clinical departments, which played a vital role in the smooth implementation of this study and the final formation of the co-management program. However, the incidence of perioperative medical complications was not further reduced by engaging emergency physicians to the MDT. Chest infections and urinary tract infections are the most common perioperative medical complications in older patients with hip fractures, with a relatively low incidence of cerebral infarction, acute coronary syndrome, DVT, and PTE (34, 35). Further study to identify problems and propose solutions for factors other than medical interventions is warranted to achieve the long-term improvement in patient’s care.

There were several limitations to this study. First, the study was designed as a retrospective study and the implementation of the two plans was not contemporaneous. Therefore, other unmeasured factors and improvement in hospital equipment may affect the accuracy of the study. Secondly, our findings might be less generalizable since all patients and project implementers were from the same hospital. To assess the ability to extent this model to other hospital and locations, we plan to conduct prospective multi-regional and multi-center studies to explore more optimized and standardized process solutions that meet the needs of China's different regions. In addition, the present study focused on investing the impact of co-management model to HF patients. Further assessment of the impact of co-management model to involved doctors, hospital staffs, as well as the cost-effectiveness of the co-management are warrant. Moreover, most of the patients were transferred from other hospitals, thus limited information of transportation before admission were collected. We hope that by strengthening pre-hospital management, increasing social awareness and education, and promoting the link between pre-hospital and post-hospital management, the growing public health problem of hip fractures in older patients can be adequately managed in our aging society.

In summary, our data showed that co-management of older hip fracture patients by emergency physicians and geriatricians is feasible and can reduce the patient’s preoperative waiting time and reduce the overall length of hospital stays. The emergency physicians participated in discussions about preoperative management as soon as each patient arrived, and also provided us with new ideas for the optimal management of older hip fracture patients from the ED to the surgical treatment. However, this intervention did not further reduce the incidence of perioperative complications and mortality during hospitalization. Therefore, we need to further explore the best management model for older patients with hip fractures.

## Data Availability

The original contributions presented in the study are included in the article/supplementary materials, further inquiries can be directed to the corresponding authors.
